# Assessing care quality in general practice: a qualitative study of GPs in Ireland

**DOI:** 10.3399/BJGPO.2023.0104

**Published:** 2024-02-21

**Authors:** Aaron Chan, Louise Hickey, Kieran Finucane, John Brennan

**Affiliations:** 1 University College Dublin School of Medicine, Dublin, Republic of Ireland; 2 Stillorgan Medical Centre, Dublin, Republic of Ireland; 3 Quality Improvement Faculty, Royal College of Physicians of Ireland, Dublin, Republic of Ireland; 4 Gowran Medical Centre, Co. Kilkenny, Dublin, Republic of Ireland

**Keywords:** general practice, quality improvement, assessment, primary healthcare

## Abstract

**Background:**

It is estimated that each year in Ireland, approximately 29 million consultations occur in general practice with a patient satisfaction level of 90%. To date, research has been lacking on how GPs assess the quality of care.

**Aim:**

To examine how GPs assess care quality during routine practice with respect to the following pillars of quality improvement: effectiveness, safety, timeliness, equity, efficiency, sustainability, and person-centredness.

**Design & setting:**

Qualitative study of GPs in Ireland.

**Method:**

In this qualitative study, semi-structured interviews were conducted with 10 GPs who were recruited via a snowball sampling strategy. Interviews were recorded, transcribed, and analysed. Quality ‘assessment points’ were identified and themes were synthesised to produce a theoretical framework.

**Results:**

Five female and five male GPs practising in a variety of settings were interviewed. The age range was 33–68 years. In total, 122 assessment points emerged from the data and were collated into the following eight themes: the GP as a professional person factors; the patient and coproduction factors; care team factors; direct care factors; outcome factors; practice environment and organisation factors; external environment factors; and improvement approach factors.

**Conclusion:**

This is the first study to examine how GPs in Ireland assess care quality as a holistic construct during daily care. The qualitative approach applied yielded rich and diverse insights into the many assessment points that GPs use to inform their approach and actions as clinicians, managers, collaborators, and leaders to maximise patient care. The theory produced is likely useful and applicable for practising GPs, healthcare administration, policymakers, and funders in planning and executing changes for quality improvement.

## How this fits in

Quality and patient safety have long been regarded as a critical aspect of patient care; however, their assessment during the day-to-day work of GPs has not been established to date. Further understanding is fundamental to ensure that quality and safety levels can be maintained and improved into the future as care needs and delivery evolve. This article has contributed to that goal as it is the first study in Ireland to examine how GPs assess care quality as a holistic construct.

## Introduction

In Ireland each year, there are approximately 29 million general practice consultations with the average person visiting their GP more than four times.^1^ Of these, roughly 90% are managed without the need for further referral.^2^ General practice plays a central role in providing comprehensive primary healthcare across communities. Patient satisfaction rates are high at 90%, with patients recognising good accessibility and person-centredness.^3^ Patients advise that a personable approach and responsive service are what really matters.^4^

These aspects of care represent key determinants of quality. Quality health care has been described as safe, timely, effective, efficient, equitable, and patient-centred.^5^ Worldwide, there is a recognised need to improve quality and safety levels for the benefit of patients and their health.^6–9^ As a means of providing high-quality health care for all, development and strengthening of primary care has been endorsed by the World Health Organization as being the most effective and efficient way of meeting the physical and mental health needs of populations.^10^ This also aligns with United Nations (UN) sustainable development goal 3.8, universal access to quality essential healthcare services.^11^

Healthcare quality can be evaluated and measured in many ways. Traditionally, this focused on objectifiable measures such as those categorised by Donabedian’s structure, process, and outcome framework.^12^ Recently, climate sustainability, wellbeing of healthcare professionals and a broader focus on ‘kinship’ have been proposed as additional elements of quality.^13–15^ The importance of culture as a perceptible determinant has also been established.^16^ Furthermore, wider assessments from the perspective of the patient, their journey, and what matters to them have been proposed.^17^

In Ireland, the Health Information and Quality Authority has published quality standards to aid quality assessment in publicly funded healthcare settings.^18^ While welcoming efforts to continuously improve healthcare quality and recognising that much within these standards is already part of established everyday care, these received mixed responses from GPs concerned by potential resource implications and bureaucratic load.^19^ Scientific and grey literature provides many examples of how specific quality domains have been and are assessed in Irish general practice including patient safety,^20–23^ equity,^24,25^ efficiency,^26,27^ and effectiveness.^28^ However, the assessment of quality as a holistic construct during the day-to-day work of GPs has not been established to date. Research from other jurisdictions suggests that additional exploration is both feasible and valuable.^29,30^ Further understanding in this area is fundamental to ensure that quality and safety levels can be maintained and improved into the future as care needs and delivery evolve.

This study aimed to address the following question: how do GPs in Ireland assess the quality of the care that they provide to their patients? This includes how clinicians measure, evaluate, quantify, and track the effectiveness, safety, timeliness, equity, efficiency, sustainability and/or person-centredness care provided during routine practice.

## Method

This research is reported using the Standards for Reporting Qualitative Research (SRQR).^31^

### Qualitative approach and research paradigm

This semi-structured interview-based research study utilised a grounded theory approach and was underpinned by a postpositivist research paradigm. This facilitated open and rich responses offering insights into the assessment of quality from the perspective of individual GPs that may not be captured by a deductive approach.^32^

The professional background and experiences of the lead researcher as a fellow GP facilitated iterative question proposition and elaboration, potentially broadening the range of responses and consequently, the richness of information revealed. As the subject area is likely to represent a significant point of pride and raises issues of professional mastery for participants, the similar background of the researcher sought to provide a psychologically safer space to explore these issues in greater depth.^33^ Analysis and interpretation of the data are framed through the subjectivity of the research team as GPs with an interest in healthcare quality and safety along with a healthcare management specialist.^34^ It is important to note that the interviewees themselves did not declare any interest in quality improvement before recruitment.

### Researchers’ characteristics and reflexivity

The lead researcher (JB) is a vocationally trained and practising GP. They have an active interest in healthcare quality improvement and patient safety. They work with teams and individuals across the Irish healthcare system to realise improvements in patient care.

The co-investigator (AC) is a third-year medical student who previously worked as a hospital quality improvement specialist, having completed an MBA specialising in health services management. They have an active interest in bridging the clinical and operational sides of health care to maximise patient care.

The two collaborating investigators are also active GPs with interests in quality and continually improving their practices.

### Context

The context for this study is general practice in Ireland, where quality and safety of care are assessed on an ongoing basis by GPs. Participants all had a mixed pool of public and private patients and therefore no discrimination or differentiation was allowed by contract.

### Sampling strategy

Non-probability sampling was utilised. A purposive maximum variation approach was adopted to recruit participants. As the lead researcher is a GP, a snowball sampling strategy was used to recruit participants nominated by a variety of colleagues but not directly known. This allowed for open, honest, and diverse responses by reducing the risk of social desirability bias.^35^ Compared with convenience sampling, this strategy also likely lead to greater variation in participation and responses.

Recruitment and further interviews ceased with data saturation.

The inclusion criteria were GPs practising clinically for a minimum of one session per week in Ireland and registered on the Irish Medical Council specialist register.

The exclusion criteria were GP trainees, GPs not practising clinically, and doctors working in general practice but not on the Irish Medical Council specialist register. Only those actively practising and who had completed training were selected to ensure adequate and up-to-date exposure to the field.

### Ethical issues

All participants provided voluntary signed informed consent, were pseudonymised at the point of interview transcription, and were assured of protection of personal data through the General Data Protection Regulation (GDPR), Data Protection Act 2018, and UK Policy Framework for Health and Social Care Research.

### Data collection methods, instruments, and technology

The study was undertaken during the COVID-19 pandemic. For convenience and health reasons, semi-structured interviews were conducted by the GP lead researcher and supported by the co-investigator medical student over Zoom between 22 July and 23 August 2022. With consent, each interview was recorded for transcription and reviewed subsequently to ensure accuracy. Interviews lasted between 34 and 62 minutes and were conducted in accordance with the interview guide (Supplementary Information S1). The questions and process were iterated with each interview and informed by previous responses. Demographic data were recorded via Google Forms at the time of consent. Data analysis was conducted between 27 August and 9 October 2022.

### Data processing

To ensure data security, recordings were saved directly from Zoom and Google Forms to a password-protected and encrypted hard-drive device. Demographic data were pseudonymised at the point of transcription. Participants were assigned a study reference number sequentially based on interview order. Transcription was performed directly and verbatim from recordings using Microsoft Word, including indicators of tone, posture, body language, and non-verbal cues.

### Data analysis

Data were analysed using an inductive approach based on grounded theory by each member of the team. Data analysis began with a familiarisation process through subdivision based on the following seven domains of healthcare quality: safety, timeliness, effectiveness, efficiency, equity, person-centredness, and sustainability. An ‘assessment point’ was deemed to be any factor identified as evaluative in nature or informing of subsequent action relating to any aspect of quality in general practice. The process of familiarisation facilitated ‘constant comparison’ and the emergence of themes from identified assessment points.^34^ Given the different backgrounds of the team, identified themes were synthesised, categorised, and collated into a taxonomy. From this, a theory was produced.

### Techniques to enhance trustworthiness

A panel consisting of five independent practising GP colleagues were invited to review the data and taxonomy to inform further iteration for enhanced truth value, to ensure consistency and optimise applicability.

A focus group was also conducted with participants to review and finalise the results. This group involved the research team, the GP panel, and four interviewees who expressed an interest at the end of their respective interviews. The objectives of the group were to assess the representativeness of the findings based on their own experiences, to identify the omission of important ideas, to ensure clarity of potential biases and methodology decisions, and to evaluate the applicability across GP settings and contexts.

## Results

### Synthesis and interpretation

Ten GPs (five female and five male) were interviewed as part of this study. The age range of participants was 33–68 years (median 44 years). All had completed a formal GP training programme. One identified as practising solely in rural settings, one in urban settings, and the remaining eight in mixed clinical settings.

In total, 122 different quality assessment points emerged from the data. These were collated into 31 sub-themes, which were categorised into eight themes, as shown in Supplementary Table S1. Empirical data to support each theme is included in Supplementary Table S2.

### Theme 1: GP as professional person factors

This theme relates to how the professional and personal characteristics of GPs are an intrinsic part of how quality is assessed in general practice. Most participants discussed formal training, postgraduate qualifications, continuing medical education, and further learning from practising as an objective means of assuring standards within the profession, especially regarding care effectiveness. More subjectively, participants reflected on a seemingly automatic process of comparison with colleagues when assessing aspects of their performance. Participants also elaborated on professional feelings, characteristics, and abilities when considering quality issues, as well as acknowledging how professional and personal aspects are important determinants of the work quality produced:

*‘Basing a lot of it on experience, both the different GPs in the practice of their current environment versus what they’ve seen previously and what their knowledge of the evidence is*.’ (GP 10, male)

*‘Being a trainer really keeps you on your toes cause you have to know the knowledge …*’ (GP 7, female)

*‘I would know myself and I’m kind of getting burned out and getting tired from it.*’ (GP 5, male)

### Theme 2: Patient and coproduction factors

All participants referenced the role of the patient in assessing care quality in general practice noting the importance of developing an open and empowering two-way therapeutic relationship. Assessments can be explicit, through direct feedback, whether informally provided during routine care or through a formal complaint. However, implicit patient motivation, understanding, knowledge, expectation, sense of empowerment, frequency of attendance, and family referrals were recognised as key aspects of assessing person-centred care. The role that the GP plays in facilitating the coproduction of health through an atmosphere conducive to collaboration and the development of longitudinal relationships was also regarded as important:

*‘It’s not just about the complaints. There are people that are generally happy as well … it helps to kind of shape what you’re going to do and how you’re gonna change things.’* (GP 9, female)

*‘... it’s very important to have the patient with you on the journey and not just be telling them what to do next ... and you have to empower the patients.*’ (GP 7, female)

*‘... it’s like a referral or a vote of confidence, if you’ve seen a family member and the next thing another family member is coming to you …*’ (GP 3, female)

### Theme 3: Care team factors

As the provision of care in general practice is team-based, participants identified assessable aspects of team arrangement and function as critical for achieving high quality care. This involved fellow physicians and all members of the work environment from administrative staff to allied health professionals. Communication between team members, in more and less structured situations, was highlighted by multiple participants as a key determinant of high quality care provision. Defined, yet flexible roles, coupled with optimal methods and mutual learning built on positive relationships, were perceived as significant. Teamwork across care boundaries was mentioned as imperative for integrating care:

*‘If staff are comfortable and enjoying work and there’s a good atmosphere and a good dynamic in the workplace, it’s, it’s bound to improve the quality of the service and it’s bound to feed down into patient care.*’ (GP 1, female)

*‘Is the thing that everyone’s doing, working at ... the highest level of their skillset?*’ (GP 2, male)

*‘We try and be proactive and listen to our staff. And we do change things regularly.*’ (GP 1, female)

### Theme 4: Direct care factors

Participants referenced many aspects of the actual process of providing direct care when assessing different aspects of care quality. This began with the varied and often complex presentation of ill health. The consultation, as both a vehicle and vessel for care provision, its timeliness, nature, length, and interruptions during which clinical acumen is exercised, was ascertained by participants as where timeliness, effectiveness, person-centredness, safety, and efficiency collide. In this unit of GP work, participants alluded to how evidence-based medicine is personalised, practice-designed protocols are followed, opportunities for further screening are realised, wider social determinants of health are explored, and occasionally errors come to pass. In the aftermath and between consultations, care continues through associated clinical administrative work, in out-of-hours and in emergency settings providing further feedback loops:

*‘Doctors get a sense … the quality we deliver by feeling that a consultation has a beginning, a middle and end, that you’ve listened, you’ve made a plan and you’ve come to the end of the consultation with the patient, happy with what you’ve done, and what you’ve discussed.’* (GP 1, female)

*‘We have protocols for typical scenarios. And those are all templated in our software so they can be used ...* ’ (GP 7, male)

### Theme 5: Outcome factors

Identified as possibly the most objective and ultimate determinant of care quality, clinical outcomes for patients, whether cure, illness control, or death are assessed continuously. This assessment can involve numerical clinical parameters or more subjective patient symptom reports. When assessing outcomes, participants implied consideration of outcomes not just for individual patients, but for families and the wider community, as well as defined geographically and by practice patient population.

*‘If they’re in with high blood pressure, you would like to see on follow-up visits that blood pressure’s controlled. You’ve got them on appropriate medication. They’re not getting side effects. They’re feeling well. Blood pressure’s a very real measurable thing*.’ (GP 4, female)

*‘if we know that, that someone has some other kind of person that they can rely on or lean on or get some kind of support from, or whatever like that family member, or perhaps even neighbours or whatever like that, you know, at least that makes it easier for us to kind of, to incorporate that into whatever kind of care we use with them.*’ (GP 8, male)

*‘I must say hard outcome measures, like morbidity, mortality, things like that, you know, and things that we can measure that against …*’ (GP 8, male)

### Theme 6: Practice environment and organisation factors

Operating also at the system level, participants discussed facets of creating and facilitating patient care for a practice overall. In assessing aspects of quality, the importance of managing access, availability, asynchronous workflows (for example, correspondence, test results, and so on) and overall capacity was voiced. The organisational challenges of staffing, balancing clinical and non-clinical work, planning for the unexpected, and limiting climate impact were also highlighted. As with any independent business, financial solvency was pointed out as a determinant of whether a practice can remain open with the necessary physical layout and infrastructure to provide care:

*‘How are we measuring access? So in simple terms, availability of appointments, availability of doctor time, availability of nurse time, delay in getting results ... how long prescription requests are sitting there.*’ (GP 1, female)

*‘It’s really important obviously to look at … the financial figures on a regular basis and make sure that things are ticking along … there’s a lot of staff that need to be paid … expenses in the practice, and then at the end of the day there has to be a few … for the doctors.*’ (GP 1, female)

*‘Physical work environments ... the rooms, the furniture, the colour of the walls, all sorts of things, really feed into a sense of wellbeing, which you know, is really important … how do we measure that? … it comes back to our meetings.*’ (GP 1, female)

### Theme 7: External environment factors

General practices operate within and are influenced by the wider health and social care system. While often not directly under the control of the GP, participants considered wider cultural changes in health care as having the potential to affect quality care in their practice either negatively (for example, defensive medicine) or positively (for example, open disclosure). This was also true of wider determinants of patient journey, such as factors affecting equity of access to general practice (funding, geography, age, ethnic group, and language). Within the tapestry of healthcare demand, supply, and oversight, wider health system access limitations, contractual obligations, workforce shortages, and national standards and protocols were evaluated by participants as particularly impactful on care effectiveness, safety, equity, and sustainability:

*‘The IMO* [Irish Medical Organisation*] have kind of figures about how ... in various counties, how many GPs were going to retire in the next five years … alarming numbers in some counties … And there doesn’t seem to be that many people around to kind of fill that role …*’ (GP 8, male)

*‘… it’s very much on clinical need … There’s no discrimination on sexual orientation or ethnicity or anything. If somebody needs an appointment and* [there is an] *appointment available, they get it.*’ (GP 4, female)

### Theme 8: Improvement approach factors

Most participants discussed the assessment of quality in the context of improving it. Several participants identified clinical audit as being a widely used structured approach to assessing quality in general practice for its improvement, together with its role in fulfilling a professional registration requirement. In addition, significant event analysis was recognised by several as an important patient safety learning approach. Outside of this, approaches to drive quality improvement included the use of quantifiable data (for example, key performance indicators [KPIs]), iterative tests of change, and formal medicines reviews. Although KPIs are direct, GPs noted how their workload influenced their ability to implement performance indicators:

*‘… it’s only by auditing really your performance, that you can learn anything … You can think you’re doing very well. You can think your outcomes are very good.*’ (GP 7, female)

*‘We’re looking at KPIs for performance on certain things, but that’s just being looked at, at the moment. There might be a little bit of resistance there …*’ (GP 7, female)

### Assessing care quality in general practice: a theoretical framework

**Figure 1.**
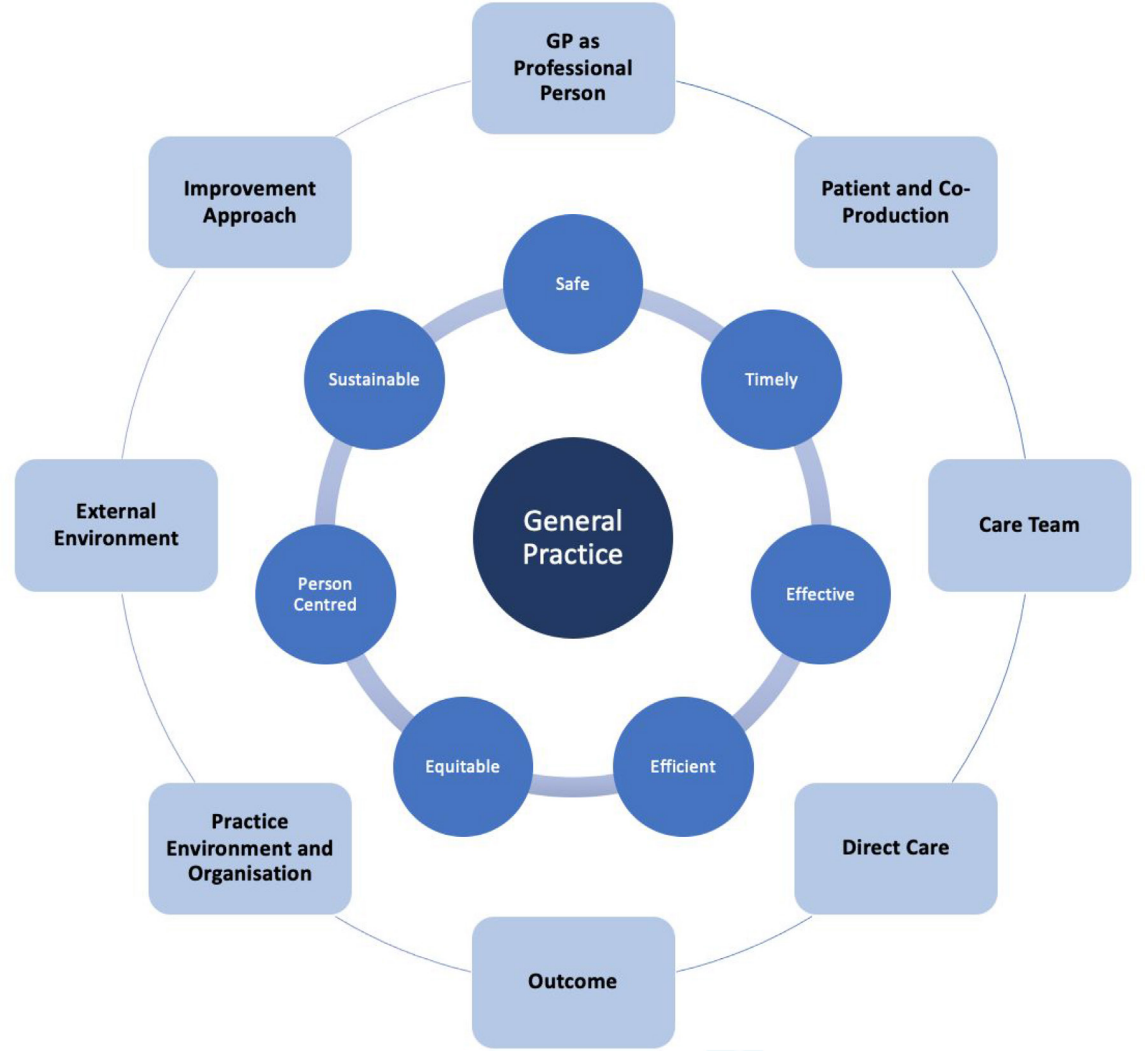
Theoretical framework of assessing care quality in general practice

Figure 1 illustrates a general theoretical framework for assessing care quality in general practice in Ireland. This theory is synthesised from the qualitative data gathered through the participant interviews, the identified quality assessment points, emergent themes, and the categorisation of these themes (Supplementary Tables S1–S2). It recognises multifaceted ways by which GPs in Ireland assess the quality of care in general practice on a comprehensive basis, both subjectively and objectively, with and without structure, measurable and unmeasurable, sometimes continuously and sometimes intermittently, clinically, organisationally, personally, and professionally.

## Discussion

### Summary

This is the first study that has examined how GPs in Ireland assess care quality as a holistic construct during routine care. The qualitative approach applied yielded rich and diverse insights into many assessment points that GPs use to inform their approach and actions as clinicians, managers, collaborators, and leaders for producing the best possible health care. Given the number and breadth of these assessment points, the emergence of themes allows the categorisation of these important factors. Although other factors, such as culture and practice-specific characteristics, need to be considered, this helps lay a foundation to guide quality improvement. The development of a theory is aimed at facilitating the pragmatic application of this new knowledge for busy GPs to assist them in better understanding and improving quality for patients. It is also likely to be of value to policymakers and funders in planning and executing change at the wider system level.

### Strengths and limitations

The strengths of this study lie in its novelty and truth value, as informed by the rich and diverse participant responses. GPs of different sexes, ages, and backgrounds were interviewed, which allowed varying perspectives to be captured. It also has immediate applicability to general practice across Ireland. By collating all quality assessment points, a single theoretical framework allowed for ease of interpretation.

There are also several limitations to this study. The sample size of 10 participants is relatively small for a qualitative study; expanding this in subsequent iterations may further improve its representativeness. Despite this, the depth and breadth of the interviews sought to mitigate this limitation. Through using a snowball sampling strategy with recruitment by colleagues, it is possible that sample variation was limited. This is evidenced by the fact that all GPs reported their ethnic group as ‘White’, and all participants had completed a formal GP training programme despite this not being an Irish Medical Council requirement to practise as a GP in Ireland.

Overall, the truth value has been optimised through iteration with an independent panel of GPs through the participation of interviewees in the focus group to review and finalise the results. There is potential that aspects of this research and theory will be applicable to similar healthcare settings outside of Irish general practice, but this remains to be tested in other contexts.

### Comparison with existing literature

The picture that has emerged from this study is one of complexity and adaptability in Irish general practice. This aligns with previously published work on systems thinking and the complex adaptive nature of health care more broadly.^36,37^ For example, in the quality domain of safety, linear cause and effect models are largely being replaced by approaches that respect health care as a socio-technical system requiring new frameworks, models, tools, and mindsets rooted in the messy real-world work of care provision.^38–40^ With these come more nuanced means of measuring and monitoring safety along a continuum, at the frontline and from different perspectives.^41^ It is necessary to appreciate the utility of this broader systems frame when faced with complexity as is evidenced here in Irish general practice; yet patient safety is only one quality domain and often different domains and ‘side effects of change’ need to be balanced.^42^

There are already examples in Ireland of successful application of this systems-based approach for quality improvement within complex organisations.^43^ The implications of these findings are timely as Irish general practice may be undergoing a rapid evolution as indicated by the recent adoption of a structured chronic disease management programme,^44^ greater access to contraception,^45^ and a move towards greater integration with other healthcare services.^46^ Further force for change is coming from an increasing burden of care as the general population ages,^47^ rising chronic disease rates,^48^ and retirement of a significant proportion of the current GP workforce.^49^ This will equate to change in the context of complexity and, consequently, quality general practice must adapt.

Context in health care can be defined as *‘a multidimensional construct encompassing micro, meso, and macro level determinants that are pre-existing, dynamic and emergent throughout the implementation process.’* ^50^ Such is the importance of context, it has been proposed that a paradigm shift in health services research may be necessary to increase the success of change implementation.^51^ Successful strides must move with this shift in laying the foundation for understanding how GPs in Ireland assess quality in their context.

A fundamental part of this context is the established frame of general practice as set out in the European definition of general practice. This emphasises many roles of the GP and the centrality of a community-oriented, comprehensive, person-centred, and holistic approach.^52^

This ethos underpins the coproduction of health, which has been defined as *‘the interdependent work of patients and professionals to design, deliver, assess, and improve the relationships and actions that contribute to the health of individuals and populations through mutual respect and partnership that leverages each participant’s unique assets, expertise, and actions*’.^53^ Coproduction has been identified as the next frontier of healthcare quality improvement, building on threshold standards and systems approaches, with demonstrable success in supporting individuals, communities, and populations in realising greater health.^54,55^ It is also intrinsic to the concept of ‘learning health systems’.^56^ As coproduction emerges so clearly from this study as a key aspect of quality assessment in general practice, it appears possible that the wider healthcare system could learn from this core focus for the improvement of other areas of health care.

### Implications for research and practice

To continuously improve healthcare quality, it is necessary to understand, in so far as is possible, how high quality health care is created. Quality is a multidimensional concept that is essential to provide safe, timely, and equitable care leading to better patient outcomes, satisfaction, and an overall improvement in the system’s efficiency and effectiveness. This study and resultant theory has demonstrated the complexity of how GPs assess quality in the dynamic setting of general practice in Ireland. This may serve as a useful guide for GPs seeking to reflect and identify areas of their own practice for improvement, as well as payers, planners, and policymakers seeking to redesign for higher quality coproduced care across populations. Further research in this area will be necessary to examine utility of this theory and to evaluate the pragmatism of identified measures as potentially useful drivers for quality improvement.

## Data

The dataset relied on in this article is available from the corresponding author on reasonable request.

## References

[1] Collins C, Homeniuk R (2021). How many general practice consultations occur in Ireland annually? Cross-sectional data from a survey of general practices. BMC Fam Pract.

[2] Gouda P, Mahambo C, Coyle E (2013). Treat or refer: factors effecting GP decisions. Forum.

[3] O’Dowd T, Ivers JH, Handy D (2017). A future together: building a better GP and primary care service.

[4] Brennan J, Bourke M, O’Donnchadha R, Mulrooney A (2019). What really matters to you? A study of public perspectives on general practice in Ireland. Ir Med J.

[5] Institute of Medicine (US) Committee on Quality of Health Care in America (2001). Crossing the quality chasm: a new health system for the 21st century.

[6] Department of Health (2020). National healthcare quality reporting system: annual report 2020..

[7] Kruk ME, Gage AD, Arsenault C (2018). High quality health systems—time for a revolution. Lancet Glob Health.

[8] World Health Organization (2018). World Health Organization, Organisation for Economic Co-operation and Development, The World Bank Delivering Quality Health Services: A Global Imperative for Universal Health Coverage.

[9] National Academies of Sciences, Engineering, and Medicine; Health and Medicine Division; Board on Health Care Services; Board on Global Health; Committee on Improving the Quality of Health Care Globally (2018). Crossing the global quality chasm: improving health care worldwide.

[10] World Health Organization (2018). Declaration of Astana.

[11] United Nations, Department of Economic and Social Affairs (2015). Sustainable Development Goals 2015..

[12] Donabedian A (1988). The quality of care. How can it be assessed?. JAMA.

[13] Mortimer F, Isherwood J, Wilkinson A, Vaux E (2018). Sustainability in quality improvement: redefining value. Future Healthc J.

[14] Sikka R, Morath JM, Leape L (2015). The quadruple aim: care, health, cost and meaning in work. BMJ Qual Saf.

[15] Lachman P, Batalden P, Vanhaecht K (2020). A multidimensional quality model: an opportunity for patients, their kin, healthcare providers and professionals to coproduce health. F1000Res.

[16] Braithwaite J, Herkes J, Ludlow K (2017). Association between organisational and workplace cultures, and patient outcomes: systematic review. BMJ Open.

[17] Hanefeld J, Powell-Jackson T, Balabanova D (2017). Understanding and measuring quality of care: dealing with complexity. Bull World Health Organ.

[18] Health Information and Quality Authority (2012). National standards for safer better healthcare.

[19] Hunter N (2015). Interpreting HIQA standards for Irish general practice. https://www.irishhealthpro.com/content/articles/show/name/interpreting-hiqa-standards-for-irish-general-practice.

[20] Madden C, Lydon S, Murphy AW, O’Connor P (2022). The patient’s “story”: an examination of patient-reported safety incidents in general practice. Fam Pract.

[21] Madden C, Lydon S, Murphy AW, O’Connor P (2021). Patients’ perception of safety climate in Irish general practice: a cross-sectional study. BMC Fam Pract.

[22] O’Dowd E, Lydon S, Lambe K (2022). Identifying hot spots for harm and blind spots across the care pathway from patient complaints about general practice. Fam Pract.

[23] Curran C, Lydon S, Kelly ME (2019). An analysis of general practitioners’ perspectives on patient safety incidents using critical incident technique interviews. Fam Pract.

[24] Nolan A, Layte R (2017). Growing up in Ireland; understanding use of general practitioner services among children in Ireland.

[25] Crowley P (2005). Health inequalities and Irish general practice in areas of deprivation. https://www.icgp.ie/speck/properties/asset/asset.cfm?type=LibraryAsset&id=39C67A51%2DC37D%2D43AA%2D85C0E6EC3EB8DB94&property=asset&revision=tip&disposition=inline&app=icgp&filename=29BB98FC%2DE7C4%2DA1F3%2DB8E1A52B531DCBD3%2Epdf.

[26] Crosbie B, O’Callaghan ME, O’Flanagan S (2020). A real-time measurement of general practice workload in the Republic of Ireland: a prospective study. Br J Gen Pract.

[27] Connolly S, Brick A, O’Neill C, O’Callaghan M (2022). An analysis of the primary care systems of Ireland and Northern Ireland. The Economic and Social Research Institute Research Series.

[28] Riordan F, McHugh S, Marsden P (2017). Audit report of the HSE Midland diabetes structured care programme.

[29] Farr M, Cressey P (2015). Understanding staff perspectives of quality in practice in healthcare. BMC Health Serv Res.

[30] Hannawa AF, Wu AW, Kolyada A (2022). The aspects of healthcare quality that are important to health professionals and patients: a qualitative study. Patient Educ Couns.

[31] O’Brien BC, Harris IB, Beckman TJ (2014). Standards for reporting qualitative research: a synthesis of recommendations. Acad Med.

[32] Pope C, Mays N (1995). Reaching the parts other methods cannot reach: an introduction to qualitative methods in health and health services research. BMJ.

[33] Edmondson AC (2018). The fearless organization: creating psychological safety in the workplace for learning, innovation, and growth.

[34] Pope C, Ziebland S, Mays N (2000). Qualitative research in health care. Analysing qualitative data. BMJ.

[35] Nederhof AJ (1985). Methods of coping with social desirability bias: a review. Eur J Soc Psychol.

[36] Peters DH (2014). The application of systems thinking in health: why use systems thinking?. Health Res Policy Syst.

[37] Braithwaite J, Ellis LA, Churruca K, Donaldson L, Ricciardi W, Sheridan S (2021). Textbook of Patient Safety and Clinical Risk Management.

[38] Carayon P, Wooldridge A, Hoonakker P (2020). SEIPS 3.0: human-centered design of the patient journey for patient safety. Appl Ergon.

[39] Holden RJ, Carayon P (2021). SEIPS 101 and seven simple SEIPS tools. BMJ Qual Saf.

[40] McNab D, McKay J, Shorrock S (2020). Development and application of ‘systems thinking’ principles for quality improvement. BMJ Open Qual.

[41] The Health Foundation (2014). A framework for measuring and monitoring safety. https://www.health.org.uk/publications/a-framework-for-measuring-and-monitoring-safety.

[42] Dixon-Woods M, McNicol S, Martin G (2012). Ten challenges in improving quality in healthcare: lessons from the Health Foundation’s programme evaluations and relevant literature. BMJ Qual Saf.

[43] Ward ME, Daly A, McNamara M (2022). A case study of a whole system approach to improvement in an acute hospital setting. Int J Environ Res Public Health.

[44] Health Service Executive (2022). Chronic disease management programme. https://www.hse.ie/eng/about/who/gmscontracts/2019agreement/chronic-disease-management-programme/.

[45] Department of Health (2022). Minister for Health launches free contraception scheme for women aged 17–25.

[46] Burke S, Barry S, Siersbaek R (2018). Sláintecare — a ten-year plan to achieve universal healthcare in Ireland. Health Policy.

[47] Central Statistics Office (2020). Projected population aged 65+..

[48] McNicholas T, Laird E (2021). Wellbeing and health in Ireland’s over 50s 2009–2016. Chapter 6: Change in chronic disease prevalence and health behaviours over the first four waves of TILDA.

[49] Irish College of General Practitioners (2018). Over 660 GPs due to retire in next 7 years as surveys show significant numbers emigrating.

[50] Rogers L, De Brún A, McAuliffe E (2020). Defining and assessing context in healthcare implementation studies: a systematic review. BMC Health Serv Res.

[51] Greenhalgh T, Papoutsi C (2018). Studying complexity in health services research: desperately seeking an overdue paradigm shift. BMC Med.

[52] World Organization of National Colleges, Academies and Academic Associations of General Practitioners/Family Physicians (WONCA) (2011). The European definition of GP/FM. https://www.woncaeurope.org/page/definition-of-general-practice-family-medicine.

[53] Batalden P (2018). Getting more health from healthcare: quality improvement must acknowledge patient coproduction—an essay by Paul Batalden. BMJ.

[54] Batalden P, Foster T (2021). From assurance to coproduction: a century of improving the quality of health-care service. Int J Qual Health Care.

[55] Batalden M, Batalden P, Margolis P (2016). Coproduction of healthcare service. BMJ Qual Saf.

[56] Gremyr A, Andersson Gäre B, Thor J (2021). The role of co-production in learning health systems. Int J Qual Health Care.

